# High temperature mortality of *Wolbachia* impacts the sex ratio of the parasitoid *Ooencyrtus mirus* (Hymenoptera: Encyrtidae)

**DOI:** 10.7717/peerj.13912

**Published:** 2022-09-13

**Authors:** Nancy R. Power, Paul F. Rugman-Jones, Richard Stouthamer, Fatemeh Ganjisaffar, Thomas M. Perring

**Affiliations:** 1 Entomology, University of California, Riverside, Riverside, CA, United States of America; 2 Rockinham, NC, United States of America; 3 Entomology and Nematology, University of California, Davis, Davis, CA, United States of America

**Keywords:** Reproductive modification, Symbiosis, *Bagrada hilaris*, Biological control

## Abstract

**Background:**

*Wolbachia* bacteria are estimated to occur in more than half of all insect species. In Hymenoptera, *Wolbachia* often manipulates its host’s reproduction to its own advantage. *Wolbachia* is likely the reason that males are rare in the uniparental *Ooencyrtus mirus* Triapitsyn & Power (Hymenoptera: Encyrtidae). The likelihood of producing male offspring can be increased by giving mothers a continuous supply of *Bagrada hilaris* (Burmeister) (Heteroptera: Pentatomidae) host eggs to parasitize for 2–3 weeks, by feeding the parents antibiotics, or by rearing parent wasps at high temperatures; all variables that have been shown to correlate with depleting *Wolbachia* titers in other organisms. The purpose of the current study was to determine whether thelytoky in *O. mirus* is due to *Wolbachia*, and if so, at what time in development the sex change occurs. We also wished to determine if *Wolbachia* removal results in the production of intersexes, as in some other hymenopterans. Finally, mating behavior was observed to see if and where it breaks down as a result of the species becoming thelytokous.

**Methods:**

Females were collected from parental lines of *O. mirus* reared at 26, 30, 31, 32, 33, 34, and 36 °C. The offspring of these females were reared at 26 °C, and their sex-ratio was determined. In a subsequent experiment, the parental generation was switched between 26 °C and 36 °C during development to narrow down the critical period at which changes occurred that subsequently affected the sex-ratio of their offspring.

**Results:**

The sex ratio was male biased in the offspring of *O. mirus* parents reared at 34 °C and 36 °C (high temperatures), even if the offspring themselves were reared at 26 °C. The constant temperature at which the percentage of males started to increase after two generations was 31 °C (10% males), rising to 39% males at 33 °C, and 100% males at 34 °C and 36 °C. Lasting more than 2 days, the critical period for the change toward a male biased sex ratio was during the second half of the parent’s development. Molecular diagnostic assays confirmed that *O. mirus* females contain *Wolbachia* and males do not. Examination of preserved males and male-female pairs under a dissecting microscope showed no signs of intersex characters. Observation of the mating behavior of live *O. mirus* showed that males initiate courtship by drumming their antennae on a female’s antennae, but after a few seconds, the females typically turn and walk away. However, a few instances of possible copulation were noted.

**Conclusions:**

As hypothesized, the results indicated that thelytoky in *O. mirus* is likely mediated by *Wolbachia* bacteria. To maximize the population growth rate without generating males, the best temperature for mass rearing this species is 30 °C.

## Introduction

*Bagrada hilaris* (Burmeister) (Heteroptera: Pentatomidae) is an invasive pest on brassica crops. Native to small parts of Africa, Asia, and the Middle East (Howard, 1906; Husain, 1925; Taylor, Bundy & McPherson, 2014), it invaded southern Europe in 1978 (Colazza, Guarino & Peri, 2004), and Los Angeles County, California, in the United States in 2008 (Arakelian, 2008). Since then, it has spread to other counties of California, and other southwestern states (Palumbo & Natwick, 2010; Bundy, Grasswitz & Sutherland, 2012; Vitanza, 2012; Perring et al., 2013; Reed et al., 2013). It also has established in Hawaii (Matsunaga, 2014), Mexico (Sánchez-Peña, 2014; Torres-Acosta & Sánchez-Peña, 2016; Hernández-Chávez et al., 2018), and Chile (Faúndez et al., 2016; Faúndez, Lüer & Cuevas, 2017) and recently in Argentina (Carpintero et al., 2021). According to Carvajal et al. (2019), its distribution has the potential of including all regions with a Mediterranean climate.

As part of an effort to identify potential biological control agents to aid in *B. hilaris* management, three hymenopteran egg parasitoid species were collected from *B. hilaris* eggs in the field in Pakistan (Mahmood et al., 2015). One of these species has recently been described as *Ooencyrtus mirus* Triapitsyn & Power (Triapitsyn et al., 2020). The biology of this new species has been under investigation to determine its potential for field release (Ganjisaffar, Power & Perring, 2021; Power, Ganjisaffar & Perring, 2020a; Power, Ganjisaffar & Perring, 2020b; Power et al., 2021).

*Ooencyrtus mirus* is a thelytokous species in which females are produced from unfertilized eggs. The current study investigates whether thelytoky in *O. mirus* is due to infection with a bacterium, *Wolbachia*. Transmitted from a female host to her offspring, *Wolbachia* are gram-negative *α*-proteobacteria (Rickettsiales: Anaplasmataceae) that are estimated to occur in more than half of all insect species (De Oliveira et al., 2015). In Hymenoptera, *Wolbachia* has been shown to manipulate its host’s reproduction in a way that favors survival of the bacterium (Stouthamer, Breeuwer & Hurst, 1999). One of the ways *Wolbachia* achieves this is by inducing parthenogenesis in which haploid eggs that would normally develop as males become diploid females. This has been documented in multiple species and results from *Wolbachia* modifying the first mitotic division in the egg, so that the resulting nucleus is diploid (Stouthamer & Kazmer, 1994; Doremus & Hunter, 2020).

If parthenogenesis in *O. mirus* is due to *Wolbachia*, then removal of the *Wolbachia* should result in a higher proportion of male offspring. *Wolbachia* can be removed in at least three ways. The first is by feeding antibiotic-laced honey to the adult female hosts (Stouthamer, Luck & Hamilton, 1990). The second is by maintaining the host colony at a temperature that is high enough to kill the *Wolbachia* but not the insects (Stevens, 1989; Stouthamer, Luck & Hamilton, 1990; Van Opijnen & Breeuwer, 1999). A third way is to provide the parasitoids a continuous supply of host eggs, which depletes a mother’s *Wolbachia* supply as she lays her eggs faster than microbe titers can recover (Lindsey & Stouthamer, 2017). In this study, we checked for the effect of *Wolbachia* removal on offspring sex ratio by rearing *O. mirus* at high temperatures. We narrowed down the temperature at which the change in sex occurs, and further narrowed down the critical developmental time period in which it occurs.

Besides increasing the proportion of males, in some species, the removal of *Wolbachia* causes intersexes to arise in some of the host offspring; *e.g.*, the sawfly *Diprion pini* (L.) (Hymenoptera: Diprionidae) (Pistone et al., 2014), the moth *Ostrinia scapulalis* (Walker) (Lepidoptera: Crambidae) (Sakamoto et al., 2008), and egg parasitoids of the genus *Trichogramma* (Hymeoptera: Trichogrammatidae) (Tulgetske & Stouthamer, 2012). Intersex individuals consist of a single sexual genotype but have both male and female sex characteristics (Tulgetske & Stouthamer, 2012).

As there are advantages of thelytokous reproduction for the application of wasps in biological control (Stouthamer & Kazmer, 1994), in some cases *Wolbachia-*infected females produce substantially fewer offspring than their arrhenotokous conspecifics. In many species of the genus *Trichogramma*, *Wolbachia*-infected thelytokous cultures can be rendered arrhenotokous by “curing” the wasps of their *Wolbachia* infection. Such cured lines often produce more female offspring than the *Wolbachia-*infected lines (Stouthamer & Luck, 1993). Here we want to determine the effects of *Wolbachia* infection and it’s removal on the offspring production of the parasitoid wasp *O. mirus* in order to determine: (1) if *Wolbachia* is the cause of thelytokous reproduction in this species; (2) if removal of the infection allows the establishment of an arrhenotokous population of this species; and (3) how rearing temperatures affect offspring sex ratios and the production of intersexes, in order to determine the best rearing temperature for this species’ application in biological control.

## Material and Methods

### Insect rearing

*Ooencyrtus mirus* and *B. hilaris* were reared according to Power, Ganjisaffar & Perring (2020a). The *O. mirus* colony was established in 2016 in the quarantine facility at the University of California, Riverside, from individuals sent from the Toba Tek Singh District in the Punjab Plain of Pakistan (Mahmood et al., 2015). The parasitoids were reared in upside-down ClickClack^®^ containers with rubber-stopper-filled holes for adding host egg cards and applying streaks of honey. These containers were maintained at room temperature of 23 °C with natural light.

The *B. hilaris* colony was started from individuals collected in Riverside, California in 2010, and refreshed annually with field-collected insects to maintain genetic diversity. The *B. hilaris* colonies were reared in tent-style cages (BugDorm^®^-2120, MegaView Science Co., Taiwan) in greenhouses set at 24−31 °C, and fed with broccoli (*Brassica oleracea* L. variety Italica), green mustard (*Brassica juncea* (L.)), and mizuna (*Brassica rapa* L. variety Japonica) seedlings grown in 10 × 10 cm plastic pots. As needed for egg collection, adults were brought to an insectary room where they were kept in round plastic containers (Durphy^®^ Packaging Co., Warminster, PA, USA) at 30 ± 1 °C, 40–50% RH and 14:10 L:D and fed organic broccoli florets. From these containers, eggs were collected daily, glued (Elmer’s^®^ Products, Inc., Columbus, OH, USA) to small pieces of card stock, and transferred to the *O. mirus* colony.

### Effect of temperature on sex ratio

To evaluate the impact of temperature on parasitoid sex ratio, *B. hilaris* host eggs were collected from the Durphy^®^ containers and glued to card stock. These eggs were provided to parasitoid females from the colony (F_0_) in nine cm height ×2 cm diameter glass vials. The vials were streaked with honey to provide food for the parasitoids. Depending on the number of eggs and the number of parasitoids available, parasitism was allowed to proceed for various amounts of time to obtain maximum parasitism, but not support superparasitism.

Typical of Encyrtidae, each *O. mirus* egg has a pedicel that protrudes through the host chorion and serves as a respiratory tube for the developing larva (Maple, 1947). A stereomicroscope was used to identify host eggs that had only one pedicel, indicating a single parasitoid egg. Each of these parasitized eggs (F_1_) was cut out on its section of card stock, placed inside a size 0 gel capsule (Capsuline^®^, Dania Beach, FL, USA), and subsequently reared at 26, 30, 31, 32, 33, 34, or 36 °C. The highest temperature (36 °C) was chosen because previous studies showed it to be the temperature at which development is quickest in *O. mirus* (Power, Ganjisaffar & Perring, 2020a). All treatments yielded 100% F1 females and these were given access to *B. hilaris* eggs to produce an F_2_ cohort, all of which were reared at 26 °C. All single-pedicel parasitized eggs were checked daily until the parasitoid emerged, at which time the sex and the total number of individuals was recorded. Data for the temperatures (31−33 °C) that yielded intermediate percentages of males (greater than 0% and less than 100%) were compared using Pearson *χ*^2^ tests in R (R Core Team, 2021).

### Critical time period for sex determination

Two separate tests were conducted to narrow down the critical period when exposure of a developing *O. mirus* female to high temperature (36° C) dramatically increased the likelihood that her progeny would be male rather than female. In the first test, 3-day-old naïve *O. mirus* females (F_0_ generation) were exposed to 1-day-old *B. hilaris* eggs glued to card stock at a ratio of 13 wasps to 25 host eggs in a glass vial for 2.25 h. The egg card was removed, and eggs with a single pedicel (F_1_ generation) were selected under a stereomicroscope and divided evenly into 7 groups. These procedures were repeated for two more days until each of the 7 groups had eleven single-pedicel eggs. Parasitized eggs from different days were kept separate. For each collection day, the vials were labeled 1 through 7, along with the date. The vial labeled as #1 was placed in a 36 °C growth chamber and the other six vials in a 26 °C growth chamber. Two days later, vial #1 was transferred to 26 °C and vial #2 was transferred to 36 °C. The same procedure was followed every subsequent 2 days, through day 12; the vial at 36 °C was returned to 26 °C, and the next sequentially numbered vial was transferred to 36 °C. All F_2_ were reared at 26.

As the total immature developmental time is 14-15 days at 26 °C (Power, Ganjisaffar & Perring, 2020a), this gave each group a chance to be at 36 °C for a different 2-day period, or 1/7 of the developmental time. When the adult wasps emerged, the number and sex were recorded. After 3 days, they were provided 1-day-old *B. hilaris* eggs at the rate of 5 host eggs per *O. mirus* female for 24 h. Again, the eggs with one pedicel (F_2_ generation) were separated out, and reared in glass vials at 26 °C until the offspring emerged. The number and sex of these F_2_ adults were recorded.

Since the F_2_ generation in the first test was 100% female (see Results), we suspected that the length of time at the high temperature was not sufficient to kill the *Wolbachia*, regardless of when the parasitoids were exposed to 36 °C. Therefore, a second test was conducted to expose the individuals for longer time periods. In this study, the F_1_ eggs were divided into four groups: (A) constant 36 °C; (B) 36 °C for four days (half of the total developmental time at 36 °C), and then transferred to 26 °C; (C) 26 °C for seven days (half the developmental time at 26 °C), and then transferred to 36 °C; and (D) constant 26 °C. Procedures for the F_2_ generation were the same as in the first critical period test, with the F_2_ generation reared at 26 °C.

### *Wolbachia* detection

In order to rule out infection with two common reproductive endosymbionts, *Cardinium* and *Rickettsia* we used established molecular assays described by Weeks, Velten & Stouthamer (2003) and Gottlieb et al. (2006), respectively. Each assay yielded a PCR product for its positive control (*Aspidiotus nerii* (Bouché) and *Bemisia tabaci* (Gennadius), respectively), but none for any of six *O. mirus* females tested. We screened for the presence of *Wolbachia* in *O. mirus* females and males using a diagnostic polymerase chain reaction (PCR) based on the bacterial 16S rRNA gene (Werren & Windsor, 2000). Females and males for these analyses were offspring of adults reared at 26 °C. Additional males were obtained by rearing parents at 36 °C, by constant exposure of *O. mirus* females to *B. hilaris* eggs for more than two weeks, and by 24-hour exposure of *O. mirus* females to *Euschistus conspersus* Uhler (Hemiptera: Pentatomidae) eggs, an alternate host of *O. mirus* (Power, Ganjisaffar & Perring, 2020b). *Ooencyrtus mirus* adults were euthanized in 95% ethanol in microcentrifuge tubes. DNA was extracted using an established Chelex^®^ resin-based method. The wasps were moved from the ethanol onto sterile filter paper to let the ethanol evaporate, after which they were placed, three per tube, into clean tubes containing 2 µl proteinase K (>600 mAU/ml; QIAGEN #19131), and ground using a sterile glass pestle. Following maceration, 60 µl of a 5% suspension of Chelex^®^ 100 (Bio Rad, Hercules, CA, USA) in water was added. The reactions were incubated in a water bath at 55 °C for 1 h, and then in a second water bath at 99 °C for 10 min. Each sample then was spun at 14,000 rpm for 4 min to pellet the Chelex^®^ resin, and 50 µl of the DNA-containing supernatant was transferred to a new tube. Extracted DNA was stored at −20 °C until used in the PCR.

Diagnostic PCRs were conducted in 25 µl volumes containing 1x Thermopol^®^ Buffer (New England Biolabs^®^, Ipswich, MA, USA), 1.5 ml BSA (10 mg/ml; New England Biolabs^®^), an additional 1 mM MgCl_2_, 0.2 mM each dNTP, 2 µl of template DNA, and 0.4 mM each of the primers W-Specf/W-Specr (Werren & Windsor, 2000). Thermocycling followed (Werren & Windsor, 2000) with the exception that we ran 42 cycles, and the extension temperature was lowered to 68 °C. The presence/absence of PCR products was checked using standard gel electrophoresis.

### Intersexuality

Forty-one males and 28 male–female pairs of *O. mirus*, all sourced from adult *O. mirus* females exposed to a continuous supply of *B. hilaris* eggs for two weeks or more, were euthanized in 95% ethanol in microcentrifuge tubes. Within each pair, the male and female were siblings that emerged on the same day in the same vial. Each preserved individual was checked under a Leica Wild M10 Stereo/Dissecting microscope for evidence of intersexuality. The features checked included setal length on both antennae, abdomen coloration, and genitalia. Male antennal setae are longer than the antennal width, whereas female setae are shorter than the antennal width. The proximal half or more of the female gaster is bright yellow, whereas the male abdomen has subtle bands of black and pale yellow (Triapitsyn et al., 2020). The female ovipositor rests in a longitudinal groove in the abdomen, whereas the male genitalia does not. The triangular tip of the ovipositor extends only slightly beyond the tip of the abdomen, whereas the male genitalia extends well past the tip of the abdomen (Triapitsyn et al., 2020).

### Mating behavior

Males and females were separated within 24 h after emergence. Three *O. mirus* males were added to a vial with three *O. mirus* females and observed under a stereoscope with fiber optics (cool) lights for 10 min. This was repeated for a total of 12 observation times with fresh males and females, each time in a clean vial. The females were 3 days old because this is the day females reach full egg laying capacity (Power et al., 2021). The males were different ages, ranging from 0 to 4 days old and at least two sets of three males of each age were observed.

## Results

### Effect of temperature on sex ratio

Female parents reared at constant 26 °C and 30 °C produced no male offspring (Table 1). A low percentage of males (2–10%) emerged from parents reared at 31 °C and 32 °C, which were not significantly different from each other (*P* = 0.098, *χ*^2^ = 2.74, *df* = 1). The percentage of males at 33 °C (39%) was significantly higher than those at 31 °C (*P <0.001, χ*^2^ = 20.17, *df* = 1) and 32 °C (*P* = 0.009, *χ*^2^ = 6.78, *df* = 1). Parents reared at 34 °C and 36 °C produced 100% male offspring. Overall, the percentage of male offspring increased from 0 to 100% as the parental rearing temperature increased from 30 °C to 34 °C (Table 1).

**Table 1:** Percentage of *Ooencyrtus mirus* female and male offspring of parents reared at different temperatures. All offspring were reared at 26 °C.

**Exposure temperature of F**_**1**_ **parents ( °C)**	**Total offspring**	**Total number of females**	**Total number of males**	**Percentage of females**	**Percentage of males**
26	129	129	0	100	0
30	35	35	0	100	0
31	29	26	3	90	10
32	51	50	1	98	2
33	46	22	14	61	39
34	28	0	28	0	100
36	73	0	73	0	100

### Critical time period for sex determination

For the first critical period test, the F1 eggs that were differentially exposed to 36 °C for 2-day intervals (labeled as 1–7) produced 47, 48, 29, 41, 39, 49, and 27 females, respectively, and 0 males. This indicated that 2 h of exposure to high temperature was not sufficient to kill the *Wolbachia* and yield male offspring. In the second test, the offspring of parents reared at constant 36 °C was 100% male (Table 2). The offspring of parents reared at 36 °C for the first half and 26 °C for the second half of their developmental time was 76% female and 24% male. The offspring of parents reared at 26 °C for the first half and 36 °C for the second half of their developmental time was 100% male. Finally, parents reared at constant 26 °C produced 100% daughters (Table 2).

**Table 2:** Second critical period test: percentage of *Ooencyrtus mirus* female and male offspring of parents reared under different temperature regimes.

**Group**	**Total Offspring**	**Total number of females**	**Total number of males**	**Percentage of females**	**Percentage of males**
A: Constant 36 °C	26	0	26	0	100
B: 36 °C then 26 °C	44	34	11	76	24
C: 26 °C then 36 °C	20	0	20	0	100
D: Constant 26 °C	25	25	0	100	0

**Notes.**

In groups B and C, the F_1_ generation was reared for the first half of their developmental time at the first temperature, and for its second half at the second temperature. The offspring (F_2_ generation) were reared at 26 °C in all four groups.

### *Wolbachia* detection

**Figure 1:**
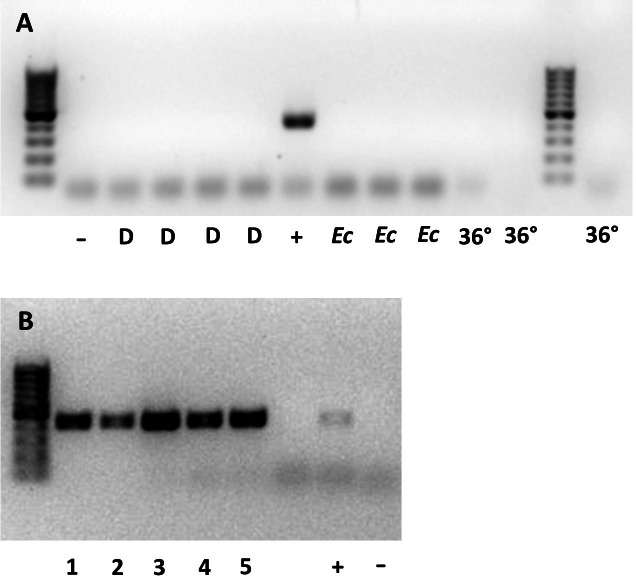
Gel plates testing for *Wolbachia* 16S rRNA gene in (A) male and (B) female *Ooencyrtus mirus*. D = depleted *Wolbachia* (males from females reared with constant supply of host eggs for more than two weeks); −= negative control; + = positive control; *Ec* = males reared on *Euschistus conspersus* host eggs, 36° = males reared at 36 °C for two generations. The numbers at the bottom of (B) represent unique samples, each containing 3 *O. mirus* females.

Whether they were sourced from 36 °C, a continuous supply of host eggs, or *E. conspersus* eggs (alternate host), all males tested negative for *Wolbachia*. None of the 10 gel electrophoresis lanes containing samples from males showed a band of the same size as the positive control for *Wolbachia* (Fig. 1A). The lanes with females all showed bands at the *Wolbachia* positive control position (Fig. 1B).

### Intersexuality

For each trait examined, all males (69 individuals) had male characters and all females (28 individuals) had female characters; no intersexuality was observed.

### Mating behavior

The most common interaction was for a male to approach a female head-on and drum his antennae on or under the female’s antennae. Within less than a second or up to 6 s, the female turned and walked away. Sometimes the male would chase the female as she walked away. On one occasion, when two males were pursuing the same female, one male tried to push the other away. In another observation, a male rocked side-to-side while antennating the female. Yet another time, two males antennated each other. Sometimes a male would pursue a female and drum his antennae on her abdomen from behind.

The newly emerged males had shorter and less frequent encounters with females than the older males did. On two different occasions, a male attempted to rear-mount a female. Nine different times, a male was observed touching the tip of his abdomen to the tip of the female abdomen for a second or two, at a 180° angle to the female with his head pointed away from her. After one of these times, the male moved backward quickly, antennae facing downward and still, and then stood in place and twitched for a few seconds, followed by grooming. The behavior preceding grooming was in stark contrast to usual male behavior, in which the males move forward, antennae facing forward and drumming rapidly.

## Discussion

### Effect of temperature on sex ratio

This study suggests that *Wolbachia* is present and active in *O. mirus* at temperatures below 34 °C. Based on the increase in males from 31−33 °C, the *Wolbachia* started to die off, or were impaired in their ability to manipulate the sex of the parasitoid offspring at these temperatures. The *Wolbachia* did not survive at 34 °C and higher. *Ooencyrtus mirus* thus tolerates higher temperatures than its symbiotic *Wolbachia*. Our sex ratio results differ from those for *Ooencyrtus submetallicus* (Howard) in Florida, USA, in which non-mated *O. submetallicus* produced males (Buschman & Whitcomb, 1980). However, our results parallel those of Wilson & Woolcock (1960) for *O. submetallicus* sourced from Trinidad Island in the Caribbean Sea, except that the critical temperature for *Wolbachia* in *O. mirus* is much higher. In *O. submetallicus*, the second generation was 100% female at 28 °C and 100% male at 29.4 °C. *Ooencyrtus mirus* had a wider transition range, with both sexes of offspring from mothers reared at 31−33 °C, and 34 °C was the lowest temperature that produced 100% males. As with *O. mirus*, the *O. submetallicus* parent generation reared at the high temperature was still mostly, or all female; only the offspring of that generation were male. The *O. mirus* - *Wolbachia* relationship is more similar to that of *Ooencyrtus pityocampae* (Merc.) in Israel. The offspring of *O. pityocampae* larvae kept at constant temperatures through 32 °C were female, with both male and female offspring arising from larvae kept at 32.5−33 °C, and only male offspring emerging from larvae kept at 34 °C or higher (Halperin, 1990). Likewise, for *Trichogramma deion* (misidentified as *T. semifumatum* (Perkins) (Hymenoptera: Trichogrammatidae) see (Pinto, 1999), but at lower temperatures, the offspring were almost all female when the parents were reared below 25.6 °C, but 97% sterile males and 3% intersexual individuals when the parents were reared at 32.2 °C (Bowen & Stern, 1966). Thelytokous *Trichogramma pretiosum* Riley have 1% male offspring at 28.26 °C and 40% males at 31 °C, whereas thelytokous *Trichogramma cordubensis* Vargas & Cabello produce 29% males at 28.26 °C and 100% males at 31 °C (Pintureauand & Bolland, 2001).

In summary, heat-killing of *Wolbachia* can occur in many hymenopteran species, but the minimum lethal temperature varies among *Wolbachia* in different host species. The minimum lethal temperature for *Wolbachia* in *O. mirus* is among the highest among those found in the literature. For all the aforementioned species, the temperatures at which F1 females are exposed during their immature development determines the sex of her F2 offspring, with high temperature leading to males and moderate temperature for each species leading to females. This implies that the eggs start to form even while the parent is a larva or pupa itself. In spite of this, however, *O. mirus* does not start ovipositing immediately after eclosion, and does not reach full oviposition capacity until 3 days after eclosion, even though the eggs laid before 3 days of age have a similar survival to eggs from parents aged 3 days and older (Power et al., 2021).

### Critical time period for sex determination

The critical time period of exposing developing *O. mirus* females to high temperature that results in males appears to be more than two days in duration, as seen by the first critical period test producing all female offspring. The critical period also seems to be toward the middle or end of the developmental time, since all of the F_2_ offspring of parents subjected to 36 °C in the second half of development were male, but only about 1/3 of the offspring of parents subjected to 36 °C in the first half of development was male. Although beyond the scope of this study, more tests with varying times at 36 °C could further narrow down the critical period. Bowen & Stern (1966), for example, narrowed down the critical period for *T. deion* to the pupal stage of the parent. The need for more than two days of high temperature to induce production of males may explain how *O. mirus* can survive (*i.e.*, not become 100% male) in the hot climate in Pakistan. Even in the hottest months, the average low temperatures are below 29 °C (Weather Spark, Excelsior, MN, USA). However, the average daytime high has a summer peak at 41 °C, so *O. mirus* may go dormant or seek out cooler microclimates during that time, unless they can endure short periods at such high temperatures. They die at a constant temperature of 38 °C in the lab (Power, Ganjisaffar & Perring, 2020a).

Halperin (1990) took the temperature transfer a step further with *O. pitycampae*. Females reared at 34 °C were transferred to 30 °C for oviposition, presumably over multiple days. First males emerged, then both males and females, and then females only. Laraichi (1978) did a similar study on *Ooencyrtus fecundus* Ferriere & Voegele. Like *O. mirus*, *O. fecundus* is an egg parasitoid of a pentatomid, in this case *Aelia cognata* Fieber. Parents were reared at 30 °C or 35 °C, and then switched to the opposite temperature after emergence. The rearing temperature determined the offspring sex for the eggs laid by the parent during her first three days of oviposition. After that, the opposite sex offspring appeared in increasing proportions. As the *O. mirus* in our study were given access to eggs for only one 24-hour period, it remains to be determined if the F_2_ sex ratio would stay the same if the females had access to eggs on subsequent days. In *O. kuvanae*, the opposite temperature effect was seen by Kamay (1976). Initial exposure to 35 °C, followed by rearing at 24 °C resulted in only 21% males, compared to 45% males reared at constant 24 °C. The effect was within the generation exposed to high temperature, not on the offspring. In this case, a different physiological mechanism than the suppression of *Wolbachia* appears to be at work.

As occurs in *Trichogramma*, the *Wolbachia* likely makes haploid eggs female by inhibiting chromosome separation during anaphase of the first round of mitosis (Stouthamer & Kazmer, 1994). *Ooencyrtus mirus* may have evolved from an arrhenotokous species that shifted to thelytoky because *Wolbachia* induced greater production of female offspring than were produced by non-infected *O. mirus* (Doremus & Hunter, 2020). In the current study, removal of *Wolbachia* did not enable *O. mirus* to return to arrhenotoky; rather it resulted in all males and thus an end to reproduction. *Wolbachia* is thus an obligate symbiont for *O. mirus*.

### *Wolbachia* detection

The absence of *Wolbachia* in the male offspring was as expected. *Ooencyrtus mirus* likely evolved from an arrhenotokous haplo-diploid species wherein unfertilized eggs became male and fertilized eggs became female. *Wolbachia* makes the haploid eggs become female. That, in turn, creates selection pressure favoring *Wolbachia*-infected individuals because females do not need to expend energy on finding mates.

In an earlier study to determine the physiological host range of *O. mirus* at 26 °C, *E. conspersus* was the only host that produced males (Power, Ganjisaffar & Perring, 2020b). Further investigation would be needed to determine whether this was by chance or if *E. conspersus* inhibits *Wolbachia* in some way.

### Intersexes

Out of 816 *O. submetallicus*, the first through fifth generation offspring reared at 21.1, 26.7, and 28.1 ° C were less than 1% males and no intersexes (Wilson & Woolcock, 1960). At 29.4 °C, however, there were 20 intersexes along with 78 males and 18 females in the second through fifth generations. In contrast, *O. mirus* had no intersexes out of 41 males and 28 male–female pairs; however our experimental set up may not have been optimal for the production of intersexes. We allowed newly emerged females to oviposit for only one day, whereas in some species infected with *Wolbachia*, intersexes are not produced on the first day of oviposition and increase in number in offspring produced in subsequent days (Tulgetske & Stouthamer, 2012).

### Mating behavior

These are the first observations of mating behavior in *O. mirus*. Compared to *O. kuvanae*, an arrhenotokous species, *O. mirus* males typically undergo only the first step of the courtship ritual, engaging his antennae with the female’s antennae. The timing of the antennation is short in *O. mirus*, usually 0–4 s and maximum 6 s before the female turns and walks away. In *O. kuvanae*, antennation lasts 4–23 s. The antennation itself is different in that *O. kuvanae* male antennae surround and lock the female antennae. In response, the female’s antennae drop and point downward. After leg strikes by the male, the female goes into a “trance” (Ablard et al., 2011). In *O. mirus*, the antennae of both sexes keep moving and no trance is induced. Earlier observations on the mating behavior of *O. kuvanae* by Alzofon (1986) included the male contacting the female with his antennae, and then walking around her until they were facing each other and touching antennae. The female would turn side-ways and the male had to follow her, remaining head-to-head, or she would walk away. In *O. kuvanae*, the male mounts the female from behind while still facing forward. In *O. mirus*, the male sometimes went tail-to-tail with the female for a few seconds. If *O. mirus* males are as quick as *Trichogramma evanescens* Westwood (Hymenoptera: Trichogrammatidae), sperm may have been transferred. In *T. evanescens*, the mean duration for the first time a male copulates is under 2 s, and the mean is under 3 s for the next seven copulations (Damiens & Boivin, 2005). In the present study, the unusual behavior of one *O. mirus* male (*i.e.*, orienting tail to tail with a female, quickly backing towards her, moving antennae downward and motionless, staying still, twitching for a few seconds, and grooming) may indicate that copulation occurred. The grooming behavior resembles that of *Cephalonomia tarsalis* (Ashmead) (Hymenoptera: Bethylidae). In this species, after the male dismounts, the two wasps separate from each other immediately, and then begin grooming (Cheng et al., 2004). In the parasitoid *Cotesia urabae* Austin & Allen (Hymenoptera: Braconidae), copulation is usually followed by stationary or grooming behavior (Avila, Withers & Holwell, 2017). Further study on *O. mirus* could show whether it is still capable of reverting to arrhenotoky, as shown in four *Trichogramma* spp. (Stouthamer, Luck & Hamilton, 1990), or whether, as in most natural populations having parthenogenesis-inducing *Wolbachia*, sexual reproduction is no longer possible (Stouthamer et al., 2010).

## Conclusions

The increase in the male: female sex ratio of *O. mirus* offspring from parents reared at high temperatures (34 °C and 36 °C), combined with the presence of *Wolbachia* in females but not in males, strongly suggests that the thelytoky in *O. mirus* is due to reproductive manipulation by *Wolbachia*. Our experiments indicate that this manipulation occurs during the second half of the parent wasp immature development. Unlike some other parasitoid species, the loss of *Wolbachia* does not cause the production of intersexes in *O. mirus*, at least in the first exposure of a naïve female to host eggs.

Although *O. mirus* develops most quickly at 36 °C (Power, Ganjisaffar & Perring, 2020a), the current study indicates that above 30 °C, the male: female ratio increases due to the depletion of *Wolbachia*. Thus, the best temperature for maximizing females in a mass rearing and release program appears to be 30 °C. Future studies are needed to ensure that the offspring continue to be mostly female in successive oviposition days and subsequent generations at this temperature.

##  Supplemental Information

10.7717/peerj.13912/supp-1 Supplemental Information 1 Data for temperature study in support of Table 1

10.7717/peerj.13912/supp-2 Supplemental Information 2 Data for critical temperature study in support of Table 2

## Additional Information and Declarations

### Data Availability

The following information was supplied regarding data availability: Raw data are available in the Supplementary Files.

## References

[ref-1] Ablard K, Fairhurst S, Andersen G, Schaefer P, Gries G (2011). Mechanisms, functions, and fitness consequences of pre-and post-copulatory rituals of the parasitoid wasp *Ooencyrtus kuvanae*. Entomologia Experimentalis et Applicata.

[ref-2] Alzofon J (1986). Biology, behavior and life table studies of *Ooencyrtus kuvanae* Howard (Hymenoptera: Encyrtidae), a gypsy moth egg parasitoid (Entomology, *Lymentria dispar*, Biological control). PhD thesis.

[ref-3] Arakelian G (2008). Bagrada bug (*Bagrada hilaris*).

[ref-4] Avila G, Withers T, Holwell G (2017). Courtship and mating behaviour in the parasitoid wasp *Cotesia urabae* (Hymenoptera: Braconidae): mate location and the influence of competition and body size on male mating success. Bulletin of Entomological Research.

[ref-5] Bowen WR, Stern VM (1966). Effect of temperature on the production of males and sexual mosaics in a uniparental race of *Trichogramma semifumatum* (Hymenoptera: Trichogrammatidae). Annals of the Entomological Society of America.

[ref-6] Bundy CS, Grasswitz TR, Sutherland C (2012). First report of the invasive stink bug *Bagrada hilaris* (Burmeister) (Heteroptera: Pentatomidae) from New Mexico, with notes on its biology. Southwest Entomologist.

[ref-7] Buschman L, Whitcomb W (1980). Parasites of *Nezara viridula* (Hemiptera: Pentatomidae) and other Hemiptera in Florida. Florida Entomologist.

[ref-8] Carpintero DL, Quiroga VN, Celentano E, Holgado MG (2021). Pimer registro de *Bagrada hilaris* (Burmeister, 1835) (Hemiptera: Pentatomidae) para la Republica Argentina. Historia Natural.

[ref-9] Carvajal MA, Alaniz AJ, Núñez Hidalgo I, González-Césped C (2019). Spatial global assessment of the pest *Bagrada hilaris* (Burmeister) (Heteroptera: Pentatomidae): current and future scenarios. Pest Management Science.

[ref-10] Cheng L-L, Howard RW, Campbell JF, Charlton RE, Nechols JR, Ramaswamy SB (2004). Mating behavior of *Cephalonomia tarsalis* (Ashmead) (Hymenoptera: Bethylidae) and the effect of female mating frequency on offspring production. Journal of Insect Behavior.

[ref-11] Colazza S, Guarino S, Peri E (2004). Bagrada hilaris (Burmeister) (Heteroptera: Pentatomidae) a pest of capper in the island of Pantelleria [*Capparis spinosa* l.; Sicily]. Informatore Fitopatologico.

[ref-12] Damiens D, Boivin G (2005). Male reproductive strategy in *Trichogramma evanescens*: sperm production and allocation to females. Physiological Entomology.

[ref-13] De Oliveira C, Gonçalves DdS, Baton LA, Shimabukuro PHF, Carvalho FD, Moreira LA (2015). Broader prevalence of *Wolbachia* in insects including potential human disease vectors. Bulletin of Entomological Research.

[ref-14] Doremus MR, Hunter MS (2020). The saboteur’s tools: common mechanistic themes across manipulative symbioses. Mechanisms Underlying Microbial Symbiosis.

[ref-15] Faúndez EI, Lüer A, Cuevas ÁG (2017). The establishment of *Bagrada hilaris* (Burmeister, 1835) (Heteroptera: Pentatomidae) in Chile, an avoidable situation?. Arquivos Entomolóxicos.

[ref-16] Faúndez EI, Lüer A, Cuevas ÁG, DA Rider, Valdebenito P (2016). First record of the painted bug *Bagrada hilaris* (Burmeister, 1835) (Heteroptera: Pentatomidae) in South America. Arquivos Entomolóxicos.

[ref-17] Ganjisaffar F, Power N, Perring TM (2021). Preferential parasitism of *Ooencyrtus mirus* (Hymenoptera: Encyrtidae) on *Bagrada hilaris* (Hemiptera: Pentatomidae) regardless of rearing host. Annals of the Entomological Society of America.

[ref-18] Gottlieb Y, Ghanim M, Chiel E, Gerling D, Portnoy V, Steinberg S, Tzuri G, Horowitz AR, Belausov E, Mozes-Daube N, Kontsedalov S, Gershon M, Gal S, Katzir N, Zchori-Fein E (2006). Identification and localization of a *Rickettsia* sp. in *Bemisia tabaci* (Homoptera: Aleyrodidae). Applied and Environmental Microbiology.

[ref-19] Halperin J (1990). Natural enemies of *Thaumetopoea* spp. (Lep. Thaumetopoeidae) in Israel 1. Journal of Applied Entomology.

[ref-20] Hernández-Chávez L, Salas-Araiza MD, Martínez-Jaime OA, Flores-Mejía S (2018). First report of *Bagrada hilaris* Burmeister, 1835 (Hemiptera: Pentatomidae) in the state of Guanajuato, Mexico. Entomological News.

[ref-21] Howard C (1906). The entomological section: the bagrada bug (*Bagrada hilaris*). Transvaal Agricultural Journal.

[ref-22] Husain M (1925). Annual report of the entomologist to government, Punjab, Lyallpur, for the year ending 30 June 1924. Report of the Department of Agriculture, Punjab.

[ref-23] Kamay B (1976). The effects of various constant temperatures on oviposition, sex ratio, and rate of development of the gypsy moth egg parasite, *Ooencyrtus kuwanai* Howard. PhD thesis.

[ref-24] Laraichi M (1978). L’effet de hautes temperatures sur le taux sexuel de *Ooencyrtus fecundus* Hymenoptera: Encyrtidae. Entomologia Experimentalis et Applicata.

[ref-25] Lindsey AR, Stouthamer R (2017). Penetrance of symbiont-mediated parthenogenesis is driven by reproductive rate in a parasitoid wasp. PeerJ.

[ref-26] Mahmood R, Jones WA, Bajwa BE, Rashid K (2015). Egg parasitoids from Pakistan as possible classical biological control agents of the invasive pest *Bagrada hilaris* (Heteroptera: Pentatomidae). Journal of Entomological Science.

[ref-27] Maple JD (1947). The eggs and first instar larvae of Encyrtidae and their morphological adaptations for respiration.

[ref-28] Matsunaga J (2014). Bagrada bug, Bagrada hilaris (Burmeister) (Hemiptera: Pentatomidae).

[ref-29] Palumbo JC, Natwick ET (2010). The bagrada bug (Hemiptera: Pentatomidae): a new invasive pest of cole crops in Arizona and California. Plant Health Progress.

[ref-30] Perring TM, Reed DA, Palumbo JC, Grasswitz T, Bundy CS, Jones W, Royer T (2013). National pest alert: bagrada bug *Bagrada hilaris* (Burmeister) family Pentatomidae.

[ref-31] Pinto JD (1999). Systematics of the North American species of *Trichogramma* (Hymenoptera: Trichogrammatidae). Memoirs of the Entomological Society of Washington.

[ref-32] Pintureauand B, Bolland P (2001). A *Trichogramma* species showing a better adaptation to high temperature than its symbionts. Biocontrol Science and Technology.

[ref-33] Pistone D, Bione A, Epis S, Pajoro M, Gaiarsa S, Bandi C, Sassera D (2014). Presence of *Wolbachia* in three hymenopteran species: *Diprion pini* (Hymenoptera: Diprionidae), Neodiprion sertifer (Hymenoptera: Diprionidae), and *Dahlbominus fuscipennis* (Hymenoptera: Eulophidae). Journal of Insect Science.

[ref-34] Power N, Ganjisaffar F, Perring TM (2020a). Effect of temperature on the survival and developmental rate of immature *Ooencyrtus mirus* (Hymenoptera: Encyrtidae). Journal of Economic Entomology.

[ref-35] Power N, Ganjisaffar F, Perring TM (2020b). Evaluation of the physiological host range for the parasitoid *Ooencyrtus mirus*, a potential biocontrol agent of *Bagrada hilaris*,. Insects.

[ref-36] Power N, Ganjisaffar F, Xu K, Perring TM (2021). Effects of parasitoid age, host egg age, and host egg freezing on reproductive success of *Ooencyrtus mirus* on *Bagrada hilaris* eggs. Environmental Entomology.

[ref-37] R Core Team (2021).

[ref-38] Reed DA, Palumbo JC, Perring TM, May C (2013). Bagrada hilaris (Hemiptera: Pentatomidae), an invasive stink bug attacking cole crops in the southwestern United States. Journal of Integrated Pest Management.

[ref-39] Sakamoto H, Kageyama D, Hoshizaki S, Ishikawa Y (2008). Heat treatment of the adzuki bean borer, Ostrinia scapulalis infected with *Wolbachia* gives rise to sexually mosaic offspring. Journal of Insect Science.

[ref-40] Sánchez-Peña SR (2014). First record in Mexico of the invasive stink bug *Bagrada hilaris*, on cultivated crucifers in Saltillo. Southwest Entomologist.

[ref-41] Stevens L (1989). Environmental factors affecting reproductive incompatibility in flour beetles, genus *Tribolium*. Journal of Invertebrate Pathology.

[ref-42] Stouthamer R, Breeuwer JA, Hurst GD (1999). Wolbachia pipientis: microbial manipulator of arthropod reproduction. Annual Reviews in Microbiology.

[ref-43] Stouthamer R, Kazmer DJ (1994). Cytogenetics of microbe-associated parthenogenesis and its consequences for gene flow in *Trichogramma* wasps. Heredity.

[ref-44] Stouthamer R, Luck RF (1993). Influence of microbe-associated parthenogenesis on the fecundity of *Trichogramma deion* and *T. pretiosum*. Entomologia Experimentalis et Applicata.

[ref-45] Stouthamer R, Luck RF, Hamilton W (1990). Antibiotics cause parthenogenetic *Trichogramma* (Hymenoptera/Trichogrammatidae) to revert to sex. Proceedings of the National Academy of Sciences of the United States of America.

[ref-46] Stouthamer R, Russell JE, Vavre F, Nunney L (2010). Intragenomic conflict in populations infected by parthenogenesis inducing *Wolbachia* ends with irreversible loss of sexual reproduction. BMC Evolutionary Biology.

[ref-47] Taylor ME, Bundy C, McPherson J (2014). Unusual ovipositional behavior of the stink bug *Bagrada hilaris* (Hemiptera: Heteroptera: Pentatomidae). Annals of the Entomological Society of America.

[ref-48] Torres-Acosta RI, Sánchez-Peña SR (2016). Geographical distribution of *Bagrada hilaris* (Hemiptera: Pentatomidae) in Mexico. Journal of Entomological Science.

[ref-49] Triapitsyn S, Andreason S, Power N, Ganjisaffar F, Fusu L, Dominguez C, Perring TM (2020). Two new species of *Ooencyrtus* (Hymenoptera, Encyrtidae), egg parasitoids of the bagrada bug *Bagrada hilaris* (Hemiptera, Pentatomidae), with taxonomic notes on *Ooencyrtus telenomicida*. Journal of Hymenoptera Research.

[ref-50] Tulgetske G, Stouthamer R (2012). Characterization of intersex production in *Trichogramma kaykai* infected with parthenogenesis-inducing *Wolbachia*. Naturwissenschaften.

[ref-51] Van Opijnen T, Breeuwer J (1999). High temperatures eliminate *Wolbachia*, a cytoplasmic incompatibility inducing endosymbiont, from the two-spotted spider mite. Experimental and Applied Acarology.

[ref-52] Vitanza S (2012). Issues in agriculture. Texas A & M AgriLife Extension. Newsletter, 38.

[ref-53] Weeks AR, Velten R, Stouthamer R (2003). Incidence of a new sex–ratio–distorting endosymbiotic bacterium among arthropods. Proceedings of the Royal Society of London. Series B: Biological Sciences, 270.

[ref-54] Werren JH, Windsor DM (2000). Wolbachia infection frequencies in insects: evidence of a global equilibrium?. Proceedings of the Royal Society of London. Series B: Biological Sciences.

[ref-55] Wilson F, Woolcock L (1960). Temperature determination of sex in a parthenogenetic parasite, Ooencyrtus submetallicus (Howard) (Hymnopera: Encyrtidae). Australian Journal of Zoology.

